# How to B(e)-1 Important Cell During *Leishmania* Infection

**DOI:** 10.3389/fcimb.2019.00424

**Published:** 2020-01-14

**Authors:** Luan Firmino-Cruz, Debora Decote-Ricardo, Daniel Claudio de Oliveira Gomes, Alexandre Morrot, Celio Geraldo Freire-de-Lima, Herbert Leonel de Matos Guedes

**Affiliations:** ^1^Laboratório de Imunofarmacologia, Instituto de Biofísica Carlos Chagas Filho, Universidade Federal Do Rio de Janeiro, Rio de Janeiro, Brazil; ^2^Laboratório Interdisciplinar de Pesquisas Médicas, Instituto Oswaldo Cruz, Fundação Oswaldo Cruz, Rio de Janeiro, Brazil; ^3^Instituto de Veterinária, Universidade Federal Rural Do Rio de Janeiro, Seropédica, Brazil; ^4^Núcleo de Doenças Infecciosas/Núcleo de Biotecnologia, Universidade Federal Do Espírito Santo, Vitoria, Brazil; ^5^Laboratório de Imunopatologia, Instituto Oswaldo Cruz, Fundação Oswaldo Cruz, Rio de Janeiro, Brazil; ^6^Faculdade de Medicina, Universidade Federal Do Rio de Janeiro, Rio de Janeiro, Brazil; ^7^Laboratório de Imunomodulação, Instituto de Biofísica Carlos Chagas Filho, Universidade Federal Do Rio de Janeiro, Rio de Janeiro, Brazil; ^8^Núcleo Multidisciplinar de Pesquisa UFRJ—Xerém em Biologia (NUMPEX-BIO), UFRJ Campus Duque de Caxias Professor Geraldo Cidade, Universidade Federal Do Rio de Janeiro, Duque de Caxias, Brazil

**Keywords:** Leishmaniasis, BALB/XID, B-1 cells, B-1CDP cells, IL-10

## Abstract

B-1 cells are an innate-like population of B lymphocytes that are subdivided into B-1a and B-1b distinguished by the presence or absence of CD5, respectively. B-1 cells can act as regulatory B cells, are able to present antigen and produce IL-10. Leishmaniasis in humans is a complex of diseases caused by parasites of the genus *Leishmania*. More than 20 species can infect humans, with each species causing the development of different immunological responses in the host. Susceptibility is usually related to the production of anti-inflammatory cytokines while the production of Th1 cytokines is indicative of resistance. However, few studies have attempted to evaluate the role of B-1 cells during either the *in vivo* infection or *in vitro* interaction with *Leishmania* parasites. *In vivo* studies were performed using XID mice model, BALB/Xid mice have a mutation in the Bruton's tyrosine kinase, which is an important enzyme for developing B-1 and maturing B-2 lymphocytes leading to the presence of immature B-2 cells. Here, we compile these studies and assess the influence of B-1 cells on disease progression with different *Leishmania* species.

## Introduction

B-1 cells are an innate-like population of B cells that are subdivided into B-1a and B-1b by the expression, or lack of, the cell marker CD5, respectively (Kantor et al., 1992; Stall et al., 1992). While the B-1a subset can be generated from precursors in the fetal liver (Tung et al., 2006), the B-1b subset is generated from precursors in the bone marrow (Tung et al., 2006) and can recognize a larger variety of antigens, including intracellular antigens (Cunningham et al., 2014). They are found mainly in the peritoneal and pleural cavities. B-1 cells have the ability to self-renew to survive long term, and have been shown to expand upon adoptive cell transfer. These cells can also secrete IgM without foreign antigen exposure (Kantor et al., 1992; Stall et al., 1992; Baumgarth, 2017), as well as naturally produce IL-10 (O'Garra and Howard, 1991).

The IL-10 production by B-1 cells was first suggested as an autocrine growth factor (O'Garra and Howard, 1991). However, a more recent study has shown that peritoneal B-1 cells from IL-10-knockout mice proliferate more than those from wild-type (WT) mice under LPS stimuli, which suggests that IL-10 could act by downregulating B-1 proliferation (Sindhava et al., 2010). It has since been speculated that the IL-10 produced by B-1 cells acts as an autocrine and paracrine regulator factor (Sindhava and Bondada, 2012).

In contrast to conventional B cells (B-2 cells), B-1 cells are able to develop immunogenic memory (Alugupalli et al., 2003; De Lorenzo et al., 2007), they can act as regulatory B cells (De Lorenzo et al., 2007) and they are also related to the innate immunity through their ability to present antigens (Vigna et al., 2006).

Parasites of the genus *Leishmania* are present worldwide with more than 20 species that can infect humans. The clinical manifestations differ from species to species, forming a complex of diseases collectively named leishmaniasis. These can be subclassified based on tissue tropism as either cutaneous leishmaniasis (CL), mucocutaneous leishmaniasis (MCL), and visceral leishmaniasis (VL). In CL, the host presents a single ulcerative lesion with swollen edges filled with parasites; however, diffuse cutaneous leishmaniasis (DCL) also can occur, where the host presents many non-ulcerative lesions filled with parasites all over the body, usually when there is pre-existing immunosuppression. In VL, also known as kalazar, the host presents high parasite burdens in the spleen and liver, and when not treated it can be fatal in 95% of the cases. Finally, MCL is characterized by disfiguring lesions in the nose and mouth area that leads to loss of the whole nose and palate.

Most of what is known about resistance or susceptibility to infections with *Leishmania* spp. is based on the host cytokine profile. While T helper (Th) type 1 lymphocyte-related cytokines are generally associated with a good prognostic (IFN-γ and TNF-α), Th2-related cytokines (IL-4, IL-5, and IL-13) and anti-inflammatory cytokines (IL-10 and TGF-β) are associated with susceptibility (Scott et al., 1989; Heinzel et al., 1991; Reiner and Locksley, 1995).

Several studies have suggested a role of B cells in promoting infection with *Leishmania* spp. either directly or indirectly via production of antibody, IL-10 or PGE_2_ (Hoerauf et al., 1994, 1995; Palanivel et al., 1996; Smelt et al., 2000; Colmenares et al., 2002; Buxbaum and Scott, 2005; Wanasen et al., 2008; Chu et al., 2010; Deak et al., 2010; Arcanjo et al., 2015, 2017a,b; Gonzaga et al., 2015, 2017; Geraldo et al., 2016). Taking CL as example, B cells are thought to be harmful to the host response. BALB/JhD, which lacks B cell (both B-1 and B-2), present lower lesions, antibodies and IL-10 than BALB/c mice when infected by *L. amazonensis* (Wanasen et al., 2008). Furthermore, in VL caused by *L. donovani* it is known that: mice which lack B cells are more resistant to infection (Smelt et al., 2000); marginal zone B cells impairs T cell responses (Bankoti et al., 2012); and the antibody production (Srinontong et al., 2018) as well as the presence of B cells (Silva-Barrios et al., 2016) are linked to pathogenesis. Besides conventional B-2 cells, B-1 cells also seem to be very important in this context (Hoerauf et al., 1994; Arcanjo et al., 2015, 2017a,b; Gonzaga et al., 2015, 2017; Geraldo et al., 2016) and here we visit several works trying to summarize the main findings in the field.

B-1 cells are related in the response to several intracellular pathogens, from opportunist infections such as microsporidia, in which they are important to control the infection upregulating T CD8^+^ cells and proinflammatory cytokines (Langanke Dos Santos et al., 2018), to parasite infections. In the present work we aimed to review the current literature regarding the participation of B-1 cells in the development of *Leishmania* spp. infections in murine models.

## The Role of B-1 Cell During *Leishmania* Major Infection

**Figure 1:**
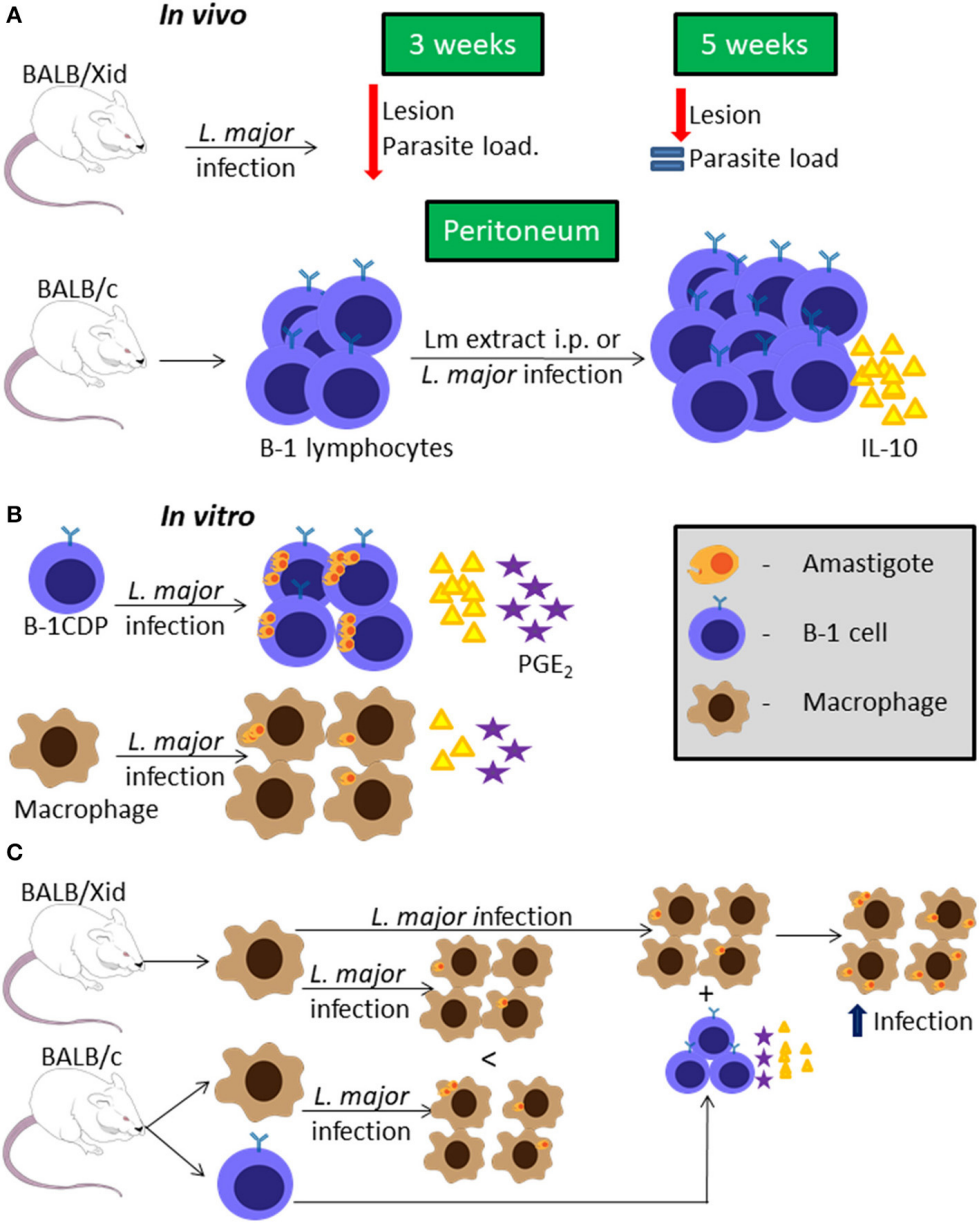
B-1 cells and *Leishmania major* infection. Representative graphic scheme about *in vivo* infection with *L. major* or stimuli with *L. major* extract **(A)**; *in vitro* infection using BALB/c cells **(B)**; or *in vitro* infection comparing BALB/c and BALB/Xid derived cells **(C)**. The schemes show a compilation of the results obtained by different research groups.

BALB/Xid mice have a mutation in the Bruton's tyrosine kinase, which is an important enzyme for developing B-1 and maturing B-2 lymphocytes (Tsukada et al., 1993) leading to the presence of immature B-2 cells (Oka et al., 1996). BALB/Xid mice infected in the footpad with *L. major* present delayed lesion development compared to WT BALB/c mice (Hoerauf et al., 1994). In addition, BALB/Xid mice have lower parasite loads at the inoculation site, draining lymph node and spleen at 3 weeks post-infection, but not at 5 weeks post-infection, compared to WT BALB/c mice (Figure 1A) (Hoerauf et al., 1994).

Peritoneal B cells (mainly B-1) were shown to produce IL-10 *in vitro*, and in the presence of *L. major* extract (Lm extract) and IL-4 stimulus per 66 h, the cells produce even more IL-10 than the non-stimulated control (Hoerauf et al., 1994). Moreover, intraperitoneal (i.p.) injection of Lm extract induces peritoneal B-1 cell proliferation and IL-10 production in BALB/c mice (Figure 1A), but not in C57BL/6 mice (Palanivel et al., 1996). However, peritoneal B-1 cells from C57BL/6 mice pre-stimulated with Lm extract i.p., when restimulated with the same extract *in vitro* are able to produce more IL-10 than the control. Peritoneal B-1 cells derived from BALB/c mice pre-stimulated with Lm extract i.p., present exacerbated IL-10 production when compared to the control (Palanivel et al., 1996). Besides that, it was shown that splenic B-1a cells are the main IL-10-producing B cell subtype during *L. major* infection, leading also to a strong Th2 signature (Ronet et al., 2010).

On the other hand, peritoneal B-1 cell-derived phagocytes (B-1CDP) are more susceptible than peritoneal macrophages to infection by *L. major in vitro*, with a higher percentage of infection, in terms of both the number of cells infected and the number of parasites per cell, as well as higher parasite proliferation (Figure 1B) (Arcanjo et al., 2015). This was attributed to the fact that B-1CDP produce more IL-10, lipid bodies and PGE_2_ endogenously than the macrophages (Figure 1B), and when the B-1CDP are treated with anti-IL-10 or non-steroidal anti-inflammatory drugs that inhibit PGE_2_ production these cells become as susceptible as macrophages (Freire-de-Lima et al., 2000, 2006; Decote-Ricardo et al., 2017). Besides that, the treatment with non-steroidal anti-inflammatory drugs decreases the level of IL-10 produced by B-1CDP and it becomes the same as the level of IL-10 produced by macrophages (Arcanjo et al., 2015). This indicates that the IL-10 production is the key factor in the susceptibility of B-1CDP cells to *L. major* infection. To further confirm this, B-1CDP from IL-10^−/−^ mice are significantly less susceptible to *L. major* than those from WT mice, with lower infection ratios and reduced parasite proliferation (Arcanjo et al., 2015).

Macrophages derived from BALB/Xid mice appear to be less susceptible to *L. major* infection than those derived from WT BALB/c mice (Figure 1C) (Arcanjo et al., 2017a). However, the presence of B-1 cells from WT BALB/c mice in the culture makes macrophages from both WT and BALB/Xid mice more susceptible to *L. major* infection *in vitro* and this phenomenon is not dependent on cell contact (Figure 1C) (Arcanjo et al., 2017a). Through the use of anti-IL-10 and non-steriodal anti-inflammatory drugs, it was again confirmed that this effect on macrophage susceptibility was due to IL-10 and PGE_2_ (Arcanjo et al., 2017a). Furthermore, the presence of B-1 lymphocytes derived from IL-10^−/−^ mice is not able to make macrophages susceptible as those derived from WT mice (Arcanjo et al., 2017a).

However, when BALB/c and C57BL/6 mice are lethally irradiated then reconstituted with autologous bone marrow, which leads to depletion of B-1 cells, there are no differences in the *L. major* disease progression between the B-1-depleted mice to their respective control, suggesting that B-1 cells may not be responsible for pathogenesis in this model (Babai et al., 1999).

## The Role of B-1 Cell During *Leishmania infantum* Infection

**Figure 2:**
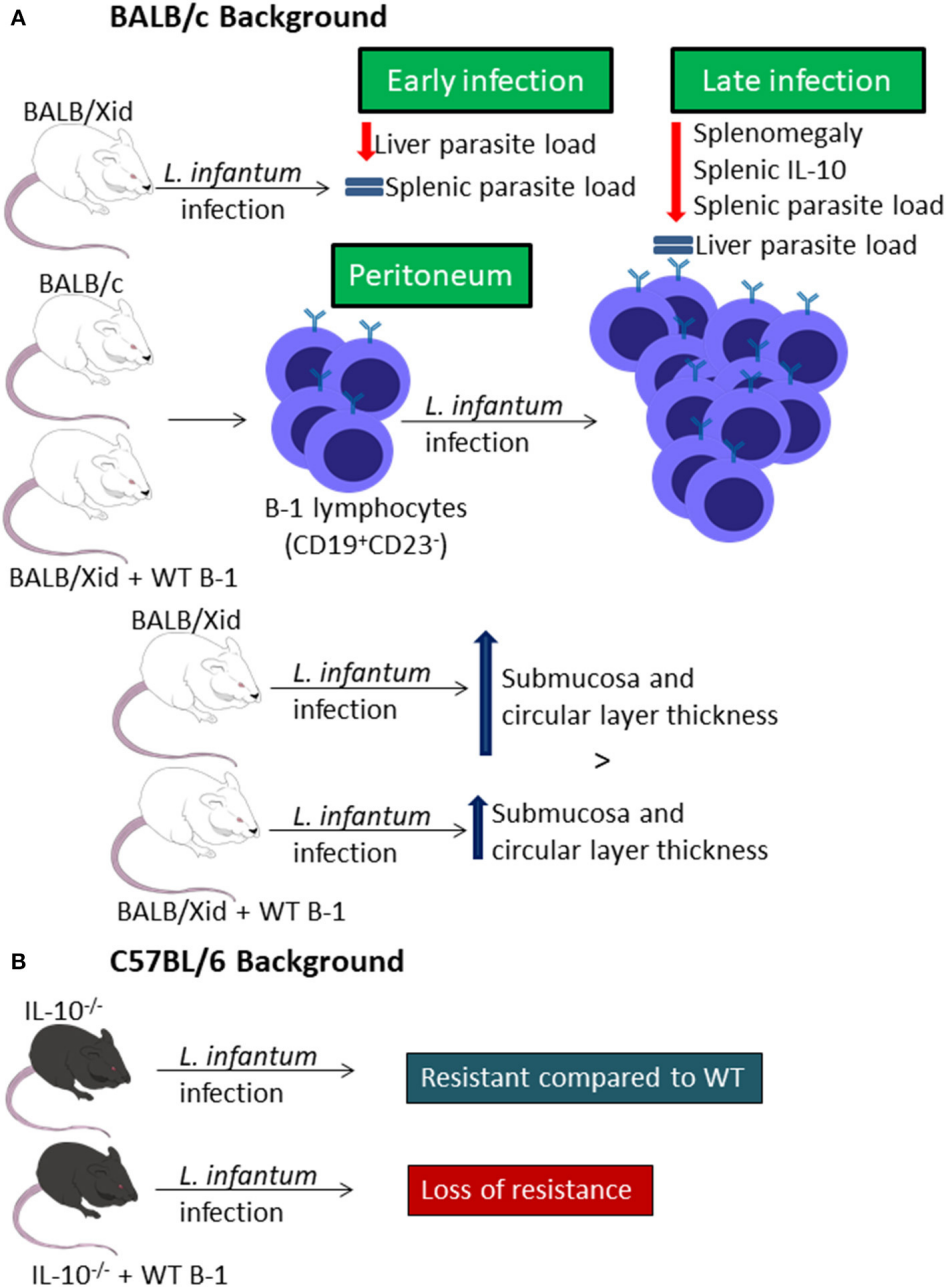
B-1 cells and *Leishmania infantum* infection. Representative graphic scheme about experimental *in vivo* infection of BALB/c **(A)** or C57BL/6 **(B)** backgrounded mice. The schemes show a compilation of the results obtained by different research groups.

In two different studies performed by two different groups, BALB/Xid mice were shown to be resistant to infection with *L. infantum* (same as *L. chagasi*), presenting lower splenomegaly and parasite loads in the spleen but not in the liver at final stages of infection (Gonzaga et al., 2015; Arcanjo et al., 2017b) probably due to lower IL-10 level in the spleen (Figure 2A) (Arcanjo et al., 2017b). However, in the early stages of infection, there is resistance of BALB/Xid mice to infection in the liver but not in the spleen (Figure 2A) (Gonzaga et al., 2015).

Infection of mice with *L. infantum* leads to an increase in the percentage of CD19^+^CD23^−^ peritoneal B cells, and the B-1 cell repopulation of BALB/Xid mice leads to loss of the resistance by these transgenic mice and to a similar profile of CD19^+^CD23^−^ peritoneal B cell (Figure 2A) (Gonzaga et al., 2015). It was also demonstrated that the infection induces differences in intestinal compartment from mice. While BALB/c mice present decrease in the thickness of the submucosa and circular layer, BALB/Xid mice present increased thickness in those sites, but the repopulation with B-1 reduces the increase index in these mice (Figure 2A) (Souza et al., 2019). Besides that, the infection also caused impaired quantitative goblet cells change, in the sialomucins and sulphomucins-producing goblet cells and in the number of Paneth cells (Figure 2A) (Souza et al., 2019).

Moreover, IL-10^−/−^ mice show resistance to *L. infantum* infection when compared to WT C57BL/6 mice, but when these mice receive an adoptive transfer of peritoneal B-1 cells they become as susceptible as WT C57BL/6 mice (Figure 2B) (Gonzaga et al., 2015).

## The Role of B-1 Cell During *Leishmania amazonensis* Infection

**Figure 3:**
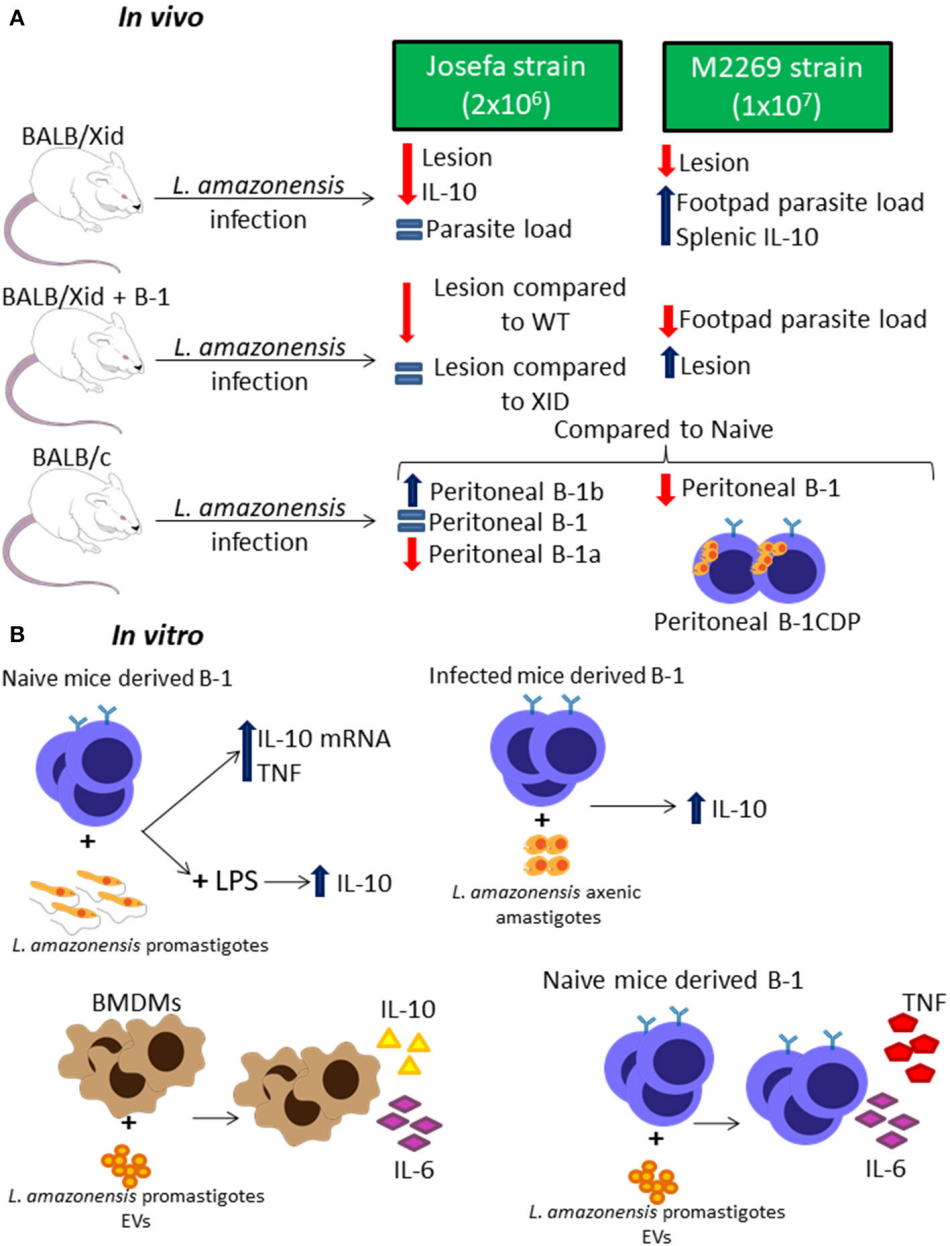
B-1 cells and *Leishmania amazonensis* infection. Representative graphic scheme about *in vivo* **(A)** and *in vitro* **(B)** infection with *L. amazonensis* parasites. The schemes show a compilation of the results obtained by different research groups.

There are a few studies around the role of B-1 cells during *L. amazonensis* infection using two different strains, the Josefa strain (Firmino-Cruz et al., 2018) and the M2269 strain (Gonzaga et al., 2017). BALB/Xid mice showed resistance in lesion growth when compared to WT BALB/c mice in both studies (Figure 3A) (Gonzaga et al., 2017; Firmino-Cruz et al., 2018). Despite the similarities in the lesion development, there is some conflicting data regarding the parasite load, as BALB/Xid mice present higher parasite load in the footpad of mice infected with the M2269 strain compared to infected WT BALB/c mice (Gonzaga et al., 2017), but there is no differences between the groups infected with the Josefa strain (Figure 3A) (Firmino-Cruz et al., 2018). While one group claims that the, when repopulated with B-1 lymphocytes, BALB/Xid mice present the same phenotype as BALB/c (Gonzaga et al., 2017), the other claims that this repopulation is not able to change BALB/Xid phenotype (Figure 3A) (Firmino-Cruz et al., 2019).

In addition, IL-10 production in these infected mice is also controversial since one group claims that BALB/Xid present a higher level of this cytokine in the spleen (Gonzaga et al., 2017), while the other shows that BALB/Xid present lower IL-10 levels in the footpads, spleen and draining lymph nodes when compared to WT BALB/c mice (Figure 3A) (Firmino-Cruz et al., 2018). It is very important to notice that the differences between these finding perhaps are related to the different parasite strain and load of infection used by each group, which can make a huge difference to the final phenotype (Loeuillet et al., 2016).

Interestingly, *L. amazonensis* infection modulates the B-1 cell profile in WT mice (Figure 3A) (Gonzaga et al., 2017; Firmino-Cruz et al., 2019). Besides that, the infection also alters the B-1 subtypes profile, increasing B-1b levels and severely decreasing B-1a levels (Figure 3A) (Firmino-Cruz et al., 2019). The interaction between peritoneal B-1 lymphocytes derived from naïve mice and *L. amazonensis* alone *in vitro* does not induce IL-10 production (Geraldo et al., 2016; Firmino-Cruz et al., 2019), even though there is an increase in the production of the mRNA of this cytokine (Figure 3B) (Geraldo et al., 2016). However, *L. amazonensis* is able to increase the IL-10 release caused by LPS and peritoneal B-1 cells derived from infected mice, when interacting to *L. amazonensis* axenic amastigotes, are able to produce more IL-10 than the ones derived from naïve mice (Figure 3B) (Firmino-Cruz et al., 2019). Moreover, the B-1 cell is able to induce the production of TNF in interaction with promastigotes (Figure 3B) (Geraldo et al., 2016). Recent studies have demonstrated that *L. amazonensis* promastigotes are able to release extracellular vesicles (EVs) which can induce bone marrow-derived macrophages (BMDMs) to increase the expression of IL-10 and IL-6, however those EVs act in B-1 cells differently, increasing IL-6 and TNF instead of IL-10 (Figure 3B) (Barbosa et al., 2018).

B-1 cells are not able to phagocytose *L. amazonensis*, however, B-1CDP cells can internalize more *L. amazonensis* parasites than peritoneal and medullar macrophages at 16 h and 24 h of infection (Geraldo et al., 2016). This phagocytic capacity was blocked by the presence of D-mannose and anti-complement receptor 3 (CR3) (Geraldo et al., 2016). B-1CDP cells can also phagocytose *L. amazonensis in vivo* (Figure 3A) (Geraldo et al., 2016).

## Concluding Remarks

In conclusion, the role of B-1 cells in infection by *Leishmania* spp. is still unclear. While a few groups were able to link pathogenesis with the presence of B-1 cells (Hoerauf et al., 1994; Arcanjo et al., 2015, 2017a,b; Gonzaga et al., 2015, 2017) other have shown that *in vivo* this is more complex (Babai et al., 1999). The fact is that the presence of *Leishmania* spp. seems to induce responses in B-1 cells, such as cytokine production (Babai et al., 1999; Arcanjo et al., 2015, 2017b; Gonzaga et al., 2015, 2017; Geraldo et al., 2016; Firmino-Cruz et al., 2019) and lipid body formation (Arcanjo et al., 2015, 2017a). However, the B-1 cell susceptibility to *Leishmania* spp. infection seems to be linked to the production of IL-10 in most of cases (Hoerauf et al., 1994; Palanivel et al., 1996; Arcanjo et al., 2015, 2017a,b; Gonzaga et al., 2015, 2017; Geraldo et al., 2016) suggesting that this cytokine promotes infection, which is not restricted to B-1 cells endogenously, but also in relation to other cells, such as macrophages (Arcanjo et al., 2017a).

There are no many papers regarding the B-1 role during infection by genus *Leishmania*. Most have been done with *Leishmania major, Leishmania amazonensis*, and *Leishmania infantum*. Besides that, there still many open questions: Can B-1 cells migrate to the lesion site during CL? Can they migrate to lymph nodes and act as APCs? How physiologic is B-1 CDP and how they act during the each infection? And most important, do they act the same way between species and strains?

More studies are still necessary to gain a complete understanding of B-1 lymphocytes during *Leishmania* spp. infection, especially because there are many species of great clinical impact that have not been checked yet.

## Author Contributions

LF-C, DD-R, DCOG, AM, CGFL and HG wrote the review. All authors read and approved the final version of the manuscript.

### Conflict of Interest

The authors declare that the research was conducted in the absence of any commercial or financial relationships that could be construed as a potential conflict of interest.
